# The primordial tRNA acceptor stem code from theoretical minimal RNA ring clusters

**DOI:** 10.1186/s12863-020-0812-2

**Published:** 2020-01-23

**Authors:** Jacques Demongeot, Hervé Seligmann

**Affiliations:** 1 Faculty of Medicine, Laboratory AGEIS EA 7407, Team Tools for e-Gnosis Medical & Labcom CNRS/UGA/OrangeLabs Telecoms4Health, Université Grenoble Alpes, F-38700 La Tronche, France; 2 The National Natural History Collections, The Hebrew University of Jerusalem, 91404 Jerusalem, Israel

**Keywords:** Teleonomy, Simulation, Origins of life, Ribosomal RNA, Translation

## Abstract

**Background:**

Theoretical minimal RNA rings code by design over the shortest length once for each of the 20 amino acids, a start and a stop codon, and form stem-loop hairpins. This defines at most 25 RNA rings of 22 nucleotides. As a group, RNA rings mimick numerous prebiotic and early life biomolecular properties: tRNAs, deamination gradients and replication origins, emergence of codon preferences for the natural circular code, and contents of early protein coding genes. These properties result from the RNA ring’s in silico design, based mainly on coding nonredundancy among overlapping translation frames, as the genetic code’s codon-amino acid assignments determine. RNA rings resemble ancestral tRNAs, defining RNA ring anticodons and corresponding cognate amino acids. Surprisingly, all examined RNA ring properties coevolve with genetic code integration ranks of RNA ring cognates, as if RNA rings mimick prebiotic and early life evolution.

**Methods:**

Distances between RNA rings were calculated using different evolutionary models. Associations between these distances and genetic code evolutionary hypotheses detect evolutionary models best describing RNA ring diversification.

**Results:**

Here pseudo-phylogenetic analyses of RNA rings produce clusters corresponding to the primordial code in tRNA acceptor stems, more so when substitution matrices from neutrally evolving pseudogenes are used rather than from functional protein coding genes reflecting selection for conserving amino acid properties.

**Conclusions:**

Results indicate RNA rings with recent cognates evolved from those with early cognates. Hence RNA rings, as designed by the genetic code’s structure, simulate tRNA stem evolution and prebiotic history along neutral chemistry-driven mutation regimes.

## Background

Several early life hypotheses assume that the first genes combined functions of structural RNAs (tRNAs, rRNAs) and protein coding genes (tRNAs and CDs [1, 2]; rRNAs and tRNAs [3–8]; rRNAs, tRNAs and CDs [9]). One of the earliest attempts to reconstruct in silico likely ancestral protein coding genes used two main principles, economy and diversity. These rational constraints mean that the sequence should code over the shortest possible length once for each genetic code signal, one start and stop codon, and each of the 20 biogenic amino acids. A third constraint, forming a stem-loop hairpin was added to delay environmental degradation [10, 11].

This defines at most 25 circular RNAs, each 22-nucleotide long, called theoretical minimal RNA rings. Surprisingly, these sequences mainly defined by coding constraints, resemble the loops of ancestral tRNAs, defining for each RNA ring an anticodon and its corresponding cognate amino acid [12]. They might also have ribozyme-like properties [13]. Hence, RNAs designed mainly according to nonredundancy among overlapping translation frames [14] recover tRNA loop sequences.

The observation that RNA rings, designed according to coding properties, coincide with tRNAs, strengthens the hypothesis of protein coding functions for tRNAs [15, 16]. This could explain biases in tRNAs (and rRNAs) for nucleotide triplets corresponding to circular code codons which are overrepresented in protein coding genes (tRNAs [17, 18]; rRNAs [19–21]). Recent analyses detect further RNA ring properties presumably characterizing prebiotic and early life sequences.

### Protein coding gene properties and RNA rings

The RNA rings have several properties that resemble those expected for actual protein coding sequences. For example, the mean position of amino acids in modern proteins, overall, resembles the order of integration of amino acids in the genetic code. Analyses showed that recent amino acids are, on average, closer to the start codon, and ancient amino acids are on average closer to the stop codon [22]. Similarly, peptides translated from RNA rings also recapitulate evolutionary orders of genetic code codon-amino acid assignments [23].

A codon property, called codon directional asymmetry (CDA), groups codons into palindromic (codon structure XYX, CDA = 0), and 5′- and 3′-extremity dominant (YXX/XXY, CDA < 0/CDA > 0). CDA > 0 associates with amino acids that are cognates of class II tRNA synthetases, CDA < 0 associates with cognates of class I tRNA synthetases [24]. Class II tRNA synthetases are believed more ancestral [25–28]. Indeed, significantly more RNA rings have more codons with CDA > 0 than with CDA < 0, as the independent hypothesis that class II tRNAs are ancestral expects [29]. Similarly, a set of 20 codons that, as a group, have mathematical properties enabling detection of the ribosomal translation frame, the near universal natural circular code [30], are overrepresented among RNA ring codons [31]. In addition, RNA ring sequences are overrepresented in ancient protein coding genes, and in particular RNA rings with ancient cognates [32].

### Replication origins and RNA rings

Natural genomes are frequently characterized by gradients in nucleotide biases, proportional to times spent single stranded during replication. This is typically due to hydrolytic deaminations, resulting in the observed nucleotide bias gradients across genomes due to replication [33–37] and/or to transcription [38, 39]. Some evidences suggest that deamination gradients do not always result from chemical changes, but from coding constraints that locate genes with high deamination risks at positions where these risks are low (i.e., close to replication/transcription origin(s)) and the genes with nucleotide contents that imply lower deamination risks at locations with higher deamination risks, i.e., at genomic locations that are distant from replication/transcription origins [36, 40].

Surprisingly, deamination gradients occur in theoretical minimal RNA rings. These overall start at the 5′ extremity of the RNA ring’s anticodon [41], as predicted by homology with ancestral tRNAs [12]. This is in line with likely homologies between mitochondrial tRNAs, their anticodon loops and loops of mitochondrial light strand replication origins (OL) [42, 43]. OL loops are the binding sites for the mitochondrial gamma DNA polymerase [44]. This polymerase and its active sites are homologues of active sites recognizing and binding the cognate tRNA anticodon loop and acceptor stem of a bacterial tRNA synthetase, the presumed ancestor of mitochondrial vertebrate gamma DNA polymerases [45–47]. Hence, deamination gradients in RNA rings start at the anticodon, the likely polymerase binding site, and reflect known homologies between tRNA synthetases and mitochondrial gamma DNA polymerase.

These evidences for RNA rings functioning as replication origins match their similarity with ancestral tRNAs [12], and the plausible origin of tRNAs from replication origin stem-loop hairpins [48].

### tRNA evolution

Previous sections show that the simple design of RNA rings implies several properties expected for ancestral multifunctional RNAs, including coding for a peptide, functioning as replication origin, and as plausible proto-tRNAs [12]. Further analyses based on RNA ring secondary structures strengthen the proto-tRNA hypothesis.

RNA secondary structures can be clustered into two main groups, presumed ancestral tRNA-like secondary structures, and presumed rRNA-like secondary structures. In rRNA-like RNAs, percentages of unpaired nucleotides within stems (forming ‘bulges’) among unpaired nucleotides, is greater than in tRNA-like RNAs. These are likely targets for enzymatic degradation and hence reflect greater regulation of RNAs forming rRNA-like secondary structures [49, 50].

The concept that rRNA-like RNAs are derived from tRNA-like RNAs was tested on tRNAs from all three kingdoms of life and from giant viruses. Cloverleaves formed by tRNAs with each of the possible cognate amino acids were ranked on the tRNA-rRNA secondary structure axis. These estimates derived from secondary structure properties were compared with the genetic code integration orders of the cognate amino acids from various hypotheses on these orders [51]. The working hypothesis expects that tRNAs with relatively more rRNA-like secondary structures have relatively recent cognate amino acids. Results overall fit this prediction, mainly in prokaryotes and viruses, confirming the evolutionary direction of the tRNA-rRNA secondary structure axis [52]. Results from similar analyses of secondary structures formed by RNA rings also follow this pattern, using cognate amino acids of predicted RNA ring anticodons. This pattern is strongest when RNA rings are spliced so as to maximize their similarity with ancestral tRNAs [53].

### RNA ring evolution?

Genetic code inclusion orders of RNA ring cognate amino acids are determined according to the RNA ring’s anticodon, which is predicted from homology with ancestral tRNA loops [12]. This inclusion order associates with each RNA ring property examined: the tRNA-rRNA axis of secondary structure of RNA rings [53], deamination gradient strengths [41], abundances of RNA ring pieces in ancient genes [32], overrepresentation of codons belonging to the natural circular code [31], overrepresentation of codons with CDA > 0 in RNA rings [29], and tendencies of amino acid sequences translated from RNA rings to recapitulate the amino acid order of integration in the genetic code [23]. Moreover, the evolutionary orders of amino acid integration hypotheses that match best with RNA ring-derived properties tend to be hypotheses derived from tRNA properties, mainly the primitive code in tRNA stems [54], tRNAs as ancestral coding genes [1, 2], and the diversity of isoacceptor tRNAs [55]. This is expected if RNA rings are proto-tRNAs.

These analyses show progressive emergence of the various properties from RNA rings with early cognates, to those with cognates having late genetic code integration ranks. This pattern is not trivial and we have no explanation for it: RNA rings result from rational in silico design, and presumably did not evolve one from the other, and/or from a common ancestor, as any evolutionary scenario would imply. Hence, we do not understand how RNA rings mimick evolutionary trends in so many properties, without any biological-historical context.

This issue is addressed here, using pseudo-phylogenetic analyses of RNA rings. In other words, do clusters of RNA rings, based on sequence similarities, associate with evolutionary hypotheses on the order of genetic code integration of their predicted cognate amino acid? This would mean that RNA rings concentrate information on the evolution of the biomolecular translation machinery. This information comes from the genetic code’s codon-amino acid assignments, which are the major information used in the design of RNA rings. This could be either because the genetic code structure embeds information related to the historical processes that formed it, and/or that its structure determines the biomolecular prebiotic and/or early life evolution.

## Results

### Distances among RNA ring sequences

The design of RNA rings is based on coding constraints. Their similarity with tRNAs is an unintended result from this design. This tRNA similarity defines the RNA ring’s anticodon and its cognate amino acid. The cognate amino acid genetic code integration order defines RNA ring evolutionary ranks. Hence, in order to understand how RNA ring comparisons reflect prebiotic and early life evolution, we focus on comparisons among RNA rings, aligning them according to their coding properties, with the stop codon at their 3′ extremity (Table 1).

**Table 1:** The 25 theoretical minimal RNA ring sequences. Columns are: 1) RNA ring numbering; 2) RNA ring cognate amino acid according to homology hypothesis with ancestral tRNAs [12]; 3) RNA ring with stop codon at 3′ extremity, anticodon underlined. Presumably, three consecutive translation rounds translated RNA rings, starting at the 3′ side of the stop codon

RNA	AA	CDs
1	F	AATTCATGCCAGACTGGTATGA
2	M	CATGCCAGAAATTCTGGTATGA
3	S	ATGGTGCCACTATTCAAGATGA
4	Pyl	ATGCTATTCACCAAGATGGTGA
5	V	ATGGTGCTACCATTCAAGATGA
6	N	ATGGCCTATTCACAAGATGTGA
7	D	ATGCCACTGGTATTCAAGATGA
8	R	ATGTGGCCTACATTCAAGATGA
9	Sec	ATGCCAAGATGGTATTCACTGA
10	L	GCAATGTTTATGGAGACCATAA
11	P	TATGTTTGGAGACCAAGCATAA
12	E	TTCATGCCAGAAACTGGTATGA
13	G	ATGGTACTGCCATTCAAGATGA
14	I	GCAGAATGTTTATGGACCATAA
15	Q	AATATGTTTGGACCAAGCATAG
16	L	TATGTTTGGAAGCCAGACATAA
17	K	TACATTTGGAAGCCAGATGTAA
18	S	ACAATGTTTATGGAAGCCATAG
19	A	ATGGAAGCCATTTACAATGTAG
20	W	TACAGATGGAAGCCATTTGTAA
21	C	AACATGCCAGATTCTGGTATGA
22	H	TGCCAGAAACATTCTGGTATGA
23	T	TATGGTTCTGCAAGAACCATGA
24	Y	TACCATTCTGCAAGAATGGTGA
25	G	ATGGTGCCATTCAAGACTATGA

The 25 RNA rings are compared using a simple distance between sequences, considering each combination of two RNA rings, aligned after splicing them at the 5′ extremity of their stop codon. Identical nucleotides at a given position have distance “0”. Non-identical ones have distance “1”. These position-specific distances are summed over the complete RNA ring length, producing pairwise distances among RNA rings. These theoretically range from “0” to “22”, because RNA rings are 22 nucleotides long. Table 2 compares RNA ring 13 and RNA ring 25. Table 3 presents the matrix of distances among all pairs of RNA rings.

**Table 2:** Distances between two RNA ring sequences, RNA ring 13 and RNA ring 25, both with predicted anticodons matching amino acid glycine (underlined). D1 is the distance between these sequences, summing the number of positions where nucleotides differ. D2 indicates a distance that accounts for mutation bias for transitions (mutations within purines (A < ->G) and within pyrimidines (C < ->T)), a distance that is more realistic in an evolutionary context. Transitions among the two sequences are indicated in italics, and are counted as distance 0.5, resulting in D2 = 8.5

RNA	AA	CDs	D1 D2
13	G	ATGGT*A*C*TGCC*ATTCAAGATGA	11 8.5
25	G	ATGGT*G*C*CATT*CAAGACTATGA

**Table 3:** D1 distances (closest to minimum, bold) among RNA rings (Table 1). Two last columns: amino acid matching predicted RNA ring anticodon/sum of D1; number N of genetic code integration hypotheses with D2 < D1/* = *P* < .05 two-tailed sign test

D1	1	2	3	4	5	6	7	8	9	10	11	12	13	14	15	16	17	18	19	20	21	22	23	24	25	AA/sum	N/test
1		**8**	16	14	16	**14**	15	16	**13**	17	13	11	15	16	14	**11**	12	16	17	**12**	10	**11**	14	16	15	F/332	41*
2	**8**		15	18	15	**14**	16	16	**13**	18	12	11	16	16	16	**11**	13	18	16	14	**8**	**8**	15	18	15	M/340	35*
3	16	15		13	**2**	**13**	**6**	**5**	**14**	15	15	11	**4**	14	15	16	18	16	**11**	20	**11**	14	14	15	**7**	S/**299**	25
4	14	18	13		11	**12**	11	12	**13**	13	16	16	10	16	16	17	16	15	**12**	15	17	18	15	**11**	10	Py/337	24
5	16	15	**2**	**11**		**12**	**6**	**5**	**14**	15	15	12	**2**	15	14	16	18	16	**13**	20	12	14	14	15	**9**	V/**299**	20
6	14	14	13	**12**	12		13	12	**13**	17	15	15	12	15	15	14	13	15	**13**	15	16	16	13	**13**	12	N/332	20
7	15	16	6	**11**	**6**	**13**		**7**	**12**	16	16	14	**4**	14	15	17	18	17	**12**	18	14	16	15	14	12	D/**316**	6*
8	16	16	**5**	**12**	**5**	**12**	**7**		15	15	16	13	6	15	15	17	18	16	**12**	18	13	16	12	**13**	11	R/**314**	12*
9	13	13	14	13	14	**13**	12	15		17	18	16	14	15	19	18	18	17	15	16	15	14	18	18	12	Sc/377	21
10	17	18	15	13	15	17	16	15	17		13	16	16	**8**	11	13	14	**4**	16	15	16	18	14	17	12	L/346	11*
11	13	**12**	15	16	15	15	16	16	18	13		14	14	12	**7**	**4**	**8**	14	17	10	15	16	**9**	**13**	16	P/318	38*
12	**11**	**11**	11	16	12	15	14	13	16	16	14		14	18	13	14	**12**	16	18	14	**4**	**8**	13	**13**	**10**	E/**316**	34*
13	15	16	**4**	**10**	**2**	**12**	**4**	**6**	**14**	16	14	14		14	15	15	17	17	**12**	18	14	16	14	15	11	G**/295**	17*
14	16	16	14	16	15	15	14	15	15	**8**	12	18	14		14	14	17	12	14	16	18	17	**11**	14	13	I/348	6*
15	14	16	15	16	14	15	15	15	19	11	**7**	13	15	14		**11**	13	**9**	17	14	12	17	**11**	14	16	Q/333	15*
16	**11**	**11**	16	17	16	**14**	17	17	18	13	**4**	14	15	14	**11**		**4**	**12**	17	**8**	14	15	**11**	15	17	L/321	36*
17	12	13	18	16	18	**13**	18	18	18	14	**8**	12	17	17	13	**4**		13	16	**4**	12	14	15	**13**	18	K/344	40*
18	16	18	16	15	16	15	17	16	17	**4**	14	16	17	12	**9**	12	13		15	15	15	18	15	18	14	S/353	22
19	17	16	11	**12**	13	**13**	12	12	15	16	17	18	12	14	17	17	16	15		16	16	17	18	17	12	A/359	9*
20	12	14	20	15	20	15	18	18	16	15	10	14	18	16	14	**8**	**4**	15	16		14	15	15	**13**	19	W/354	32*
21	**10**	**8**	11	17	12	16	14	13	15	16	15	**4**	14	18	12	14	12	15	16	14		**7**	15	15	11	C/**314**	27
22	**11**	**8**	14	18	14	16	16	16	**14**	18	16	**8**	16	17	17	15	14	18	17	15	7		17	15	15	H/352	37*
23	14	15	14	15	14	**13**	15	12	18	14	9	13	14	**11**	**11**	**11**	15	15	18	15	15	17		**6**	13	T/327	29
24	16	18	15	**11**	15	**13**	14	13	18	17	13	13	15	14	14	15	13	18	17	13	15	15	**6**		16	Y/347	36*
25	15	15	7	**10**	9	**12**	12	11	**12**	**12**	16	**10**	11	**13**	16	17	18	14	**12**	19	**11**	15	13	16		G/**316**	24

**Fig. 1:**
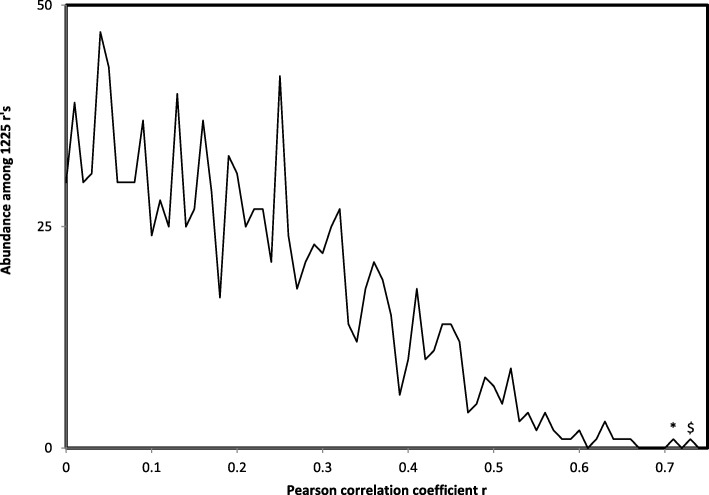
Abundance of absolute values of Pearson correlation coefficients between distances among RNA ring sequences and hypotheses on amino acid integration order in the genetic code. The distribution of r is discontinuous between 0.66 and 0.71, with two r’s above 0.71 between distances to RNA ring 3 and i) the genetic code integration order derived from the GNN hypothesis (*, *r* = 0.72, two tailed *P* = 0.00007), and ii) the primitive tRNA stem code ($, *r* = 0.733, two tailed *P* = 0.00005)

The distance matrix in Table 3 is analyzed for associations with genetic code integration orders of RNA ring cognate amino acids [51], as these are determined by the RNA ring anticodon, defined by homology with ancestral tRNAs (Table 1). Analyses consider separately distances to each focal RNA ring with the remaining 24 RNA rings. The frequency distribution of these Pearson correlation coefficients shows a clear high correlation outlier (Fig. 1): the association between distances to RNA ring 3 (anticodon corresponding to cognate Ser) and the presence of a primordial code in the tRNA acceptor stem of some tRNAs with presumed ancient cognate amino acids. These consist of prokaryote (Archaea and Bacteria) tRNAs for cognates A, D, G, V, in which the 5′ acceptor stems have at positions 3–5 nucleotide triplets coding for the amino acid that is the tRNA’s cognate amino acid [54]. The average distance between RNA ring 3 and RNA rings with predicted cognates A, D, G or V, as compared to the average distance to the remaining RNA rings is statistically significant (6 ± 3.39 vs 14.21 ± 3.07, *P* = 0.00003, two tailed t-test). After Bonferroni correction considering the 1225 correlations calculated, this corresponds to *P* = 0.03763, which is still statistically significant at *P* < 0.05.

Note that the genetic code integration hypothesis that associates best with distances among RNA rings is one among the three tRNA-derived hypotheses. This is in line with the RNA ring proto-tRNA hypothesis.

### Simple evolutionary distances among RNA rings

The previous section shows that RNA ring clusters match a primitive code in tRNA stems. This raises the question whether distances among RNA rings could be interpreted in an evolutionary sense, considering that RNA rings are rational constructs, and a priori did not evolve one from the other by point mutations. Nucleotide sequence evolution is characterized by a strong bias for transitions, meaning purine-to-purine and pyrimidine-to-pyrimidine mutations ((A < ->G and C < ->T, respectively), as opposed to transversions, purine-to-pyrimidine and pyrimidine-to-purine point mutations (the eight remaining point mutation types, A < ->C, A < ->T, C < ->G and G < ->T). Transitions are more frequent, reflecting on average shorter evolutionary distances between sequences. Hence, when differences between two RNA rings could be interpreted as due to transitions, distances are set to “0.5”, while when these are due to transversions, distances remain as previously “1”. Table 4 presents D2, the evolutionary distance matrix among RNA rings.

**Table 4:** D2 evolutionary distances among RNA rings. Transversions, as D1; distance 0.5 for transitions. Bold, closest to minimum

D2	1	2	3	4	5	6	7	8	9	10	11	12	13	14	15	16	17	18	19	20	21	22	23	24	25
1		**8**	13.5	11	13.5	11.5	12.5	13.5	10.5	14	11.5	10	12.5	13.5	11.5	10	10.5	13	13	**10**	9.5	10	12	13.5	12.5
2	**6.5**		12.5	15	12.5	12	13.5	13.5	**10**	15	9.5	**9.5**	13.5	13	13	8.5	10	15	12.5	10.5	**7**	**7**	12.5	15	12.5
3	14.5	13.5		11.5	2	**11**	**5.5**	**5**	12.5	13.5	13	**9.5**	3.5	12.5	13.5	13.5	15	14	**9**	17	10.5	13	12.5	13	**6.5**
4	12	15	11		9	**10**	9.5	10	**11**	**10**	13	13	8.5	13.5	13	13.5	13	11.5	**10**	13	14.5	15	12.5	**9.5**	8.5
5	14	12.5	**1.5**	9.5		**10.5**	**6**	**4.5**	11.5	13	13	10	**2**	13	12.5	13.5	15	13.5	10.5	16.5	10	11.5	12.5	13	**7.5**
6	12	12	11.5	10	10.5		11.5	10.5	**10.5**	13.5	12	13	10.5	12	12	11.5	11	12	10.5	12	14	13.5	10.5	11	9.5
7	13.5	13.5	5.5	9.5	5.5	11.5		**6.5**	**10**	13.5	14	12	**4**	12.5	13	14.5	15	14	**10**	15	12	13.5	13.5	12	10.5
8	14	13.5	**4.5**	**10.5**	4.5	**11**	**5.5**		13	12.5	13.5	12	**5**	12.5	13	14	15	13.5	**9.5**	15	12	14	11	12	10
9	11.5	10.5	12	11	12	**10.5**	10.5	13		14	14.5	13	12.5	12.5	15.5	15	15	14	12.5	14	13	11.5	15	15	9.5
10	14	14.5	12.5	10	12.5	13	14	12	14		10.5	12.5	13.5	**7**	**8.5**	10.5	11	**3**	13	12	12.5	14.5	11.5	13.5	10
11	10	9	12.5	12.5	12.5	12	13.5	13.5	14.5	**10.5**		11.5	11.5	**10**	**6**	**2**	**5.5**	10.5	13.5	**7**	12	13	**7**	**10.5**	13
12	**9.5**	9.5	9.5	13	10.5	13	12	11.5	13	13.5	12.5		12	15.5	11	12	9.5	13.5	14	11	**3**	**7.5**	11.5	11	**8**
13	13.5	13.5	**3**	**9**	**1.5**	**10**	**4**	**5**	12	14	12	12		12.5	13.5	12	13.5	14.5	**9.5**	15	12	13.5	12.5	13	9.5
14	12.5	12	11.5	12.5	12	**11**	12	12	11.5	**7**	9.5	14.5	11.5		11	10.5	13	**9.5**	11	12.5	14	13	8.5	**10.5**	10.5
15	11	13	12	12.5	11.5	**11**	12.5	12	15	8.5	**6**	10	12.5	11.5		8	9.5	**6.5**	14	10.5	9	13.5	8.5	11	12.5
16	10	9.5	13	13	13	**11**	14	14	15	**10.5**	2	11.5	12	11.5	**8**		**3.5**	**9.5**	13.5	6.5	12	12.5	**8**	11.5	13.5
17	**9.5**	10	14.5	13	14.5	**11**	14.5	14.5	15	11.5	**6.5**	**9.5**	13.5	14	10	**3.5**		10.5	13	**3**	9	11.5	12.5	11	14.5
18	14	15.5	14	12	14	12.5	14.5	14	14.5	2.5	11.5	14	14.5	10	8	10	10.5		13	12	13.5	16	12.5	14.5	12.5
19	13.5	12.5	8.5	9.5	9.5	**10**	9.5	9	12	13	13.5	15	9	11.5	14	13.5	13	12.5		13.5	13	13.5	14.5	14	9.5
20	**9.5**	11	16	12.5	16	11.5	14.5	14	13	11.5	7.5	11	14.5	13	10.5	**6**	**2.5**	11	13		11	12	12	11	15
21	**9**	**7**	9	13.5	9.5	13	11	11	11.5	13	12.5	**3.5**	11	14.5	9.5	12	9.5	12	12	11		**6**	13	12.5	9
22	**9.5**	**7.5**	12.5	15.5	12.5	14	14.5	14.5	12	15.5	13.5	**6.5**	14.5	15	14	13	11.5	15.5	14.5	12	**6.5**		15	12.5	13
23	13	13.5	13	13.5	13	**11**	14	11	15.5	12	8	12	13	**9.5**	9.5	9	12.5	12	15	12.5	13.5	15		**5.5**	12
24	14	15.5	13	**10**	13	**11**	12	11	15.5	14.5	11.5	11.5	13	11.5	11	13	11.5	14.5	14.5	11	12.5	13	**5.5**		14.5
25	15	15	7	**10**	9	12	12	11	12	12	16	10	11	13	16	17	18	14	12	19	11	15	13	16	

The hypothesis that RNA rings evolved from each other predicts that associations with genetic code inclusion hypotheses for RNA ring cognate amino acids should be stronger for D2 than D1. This is not the case: only 50.37% of all comparisons between associations of genetic code inclusion hypotheses with D1 vs D2 indicate stronger associations with D2. For three genetic code inclusion hypotheses, significant majorities of associations were stronger for D2 than D1 (more than 17 cases among 25 comparisons for each hypothesis): Fox’s proteinoid hypothesis [56], yields from Bar Nun’s shock wave experiment of [57], and the tRNA stem primitive code [54]. Notably, the difference between the outliers and the bulk of the distribution of r values from Fig. 1 becomes more extreme, as the strongest correlation (with the tRNA stem primitive code hypothesis) increases from r = 0.733 to r = 748. The average distance between RNA ring 3 (cognate amino acid S) and RNA rings predicted cognates A, D, G or V, as compared to the average distance to the remaining RNA rings is statistically significant (5.1 ± 2.86 vs 12.0 ± 2.42, *P* = 0.00002, two tailed t-test). After Bonferroni correction considering the 1225 correlations calculated, this corresponds to *P* = 0.02050, which is still statistically significant at *P* < 0.05.

For specific RNA rings, D1 yields significantly more stronger associations than D2 for 7 RNA rings (7,8,10,13,14,15 and 19, cognates D, R, L, G, I, Q and A), and vice versa for 9 RNA rings (1,2,11,12,16,17,20,22,24, cognates F, M, P, E, L, K, W, H and Y), according to two tailed sign tests. RNA rings with recent cognate amino acids have more genetic code integration order hypotheses for which D2 produces stronger associations than D1 than presumably more ancient RNA rings in 91.8% of the genetic code integration hypotheses examined. Effects are strongest for the RNY hypothesis [58], the Jiménez-Montaño hypothesis [59], and the tRNA stem primitive code [54]. Note that associations within tRNA structures were very early observed as reflecting the evolution of the genetic code [60]. Hence, variation among RNA rings can be seen as evolutionary, meaning resulting from point mutations that transform one RNA ring into another, mainly for recent RNA rings.

### RNA ring evolution with observed nucleotide substitutions

**Fig. 2:**
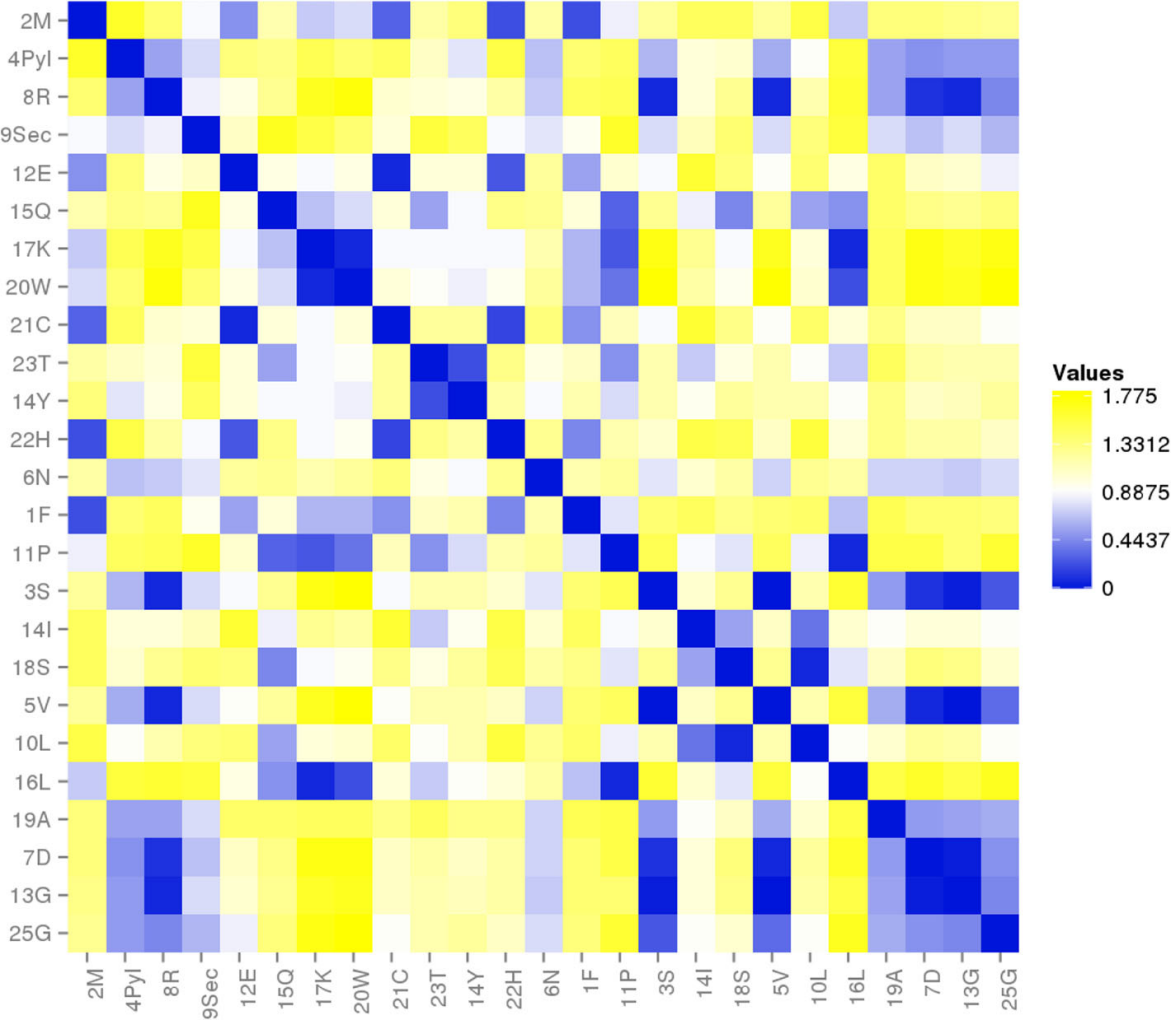
Heatmap of pairwise D3 distances among 25 RNA rings, ordered by increasing rank of mean percentage of tRNAs (with the same cognate amino acid as the RNA ring) with primitive code in their acceptor stem (averaged across kingdoms as per data in [54]). The tRNAs considered in [54] with primitive acceptor stem code have cognates A, D, G, V. The heatmap shows these clusters (blueish colors) with RNA rings 3, 4, 8 and 9 (cognates S, Pyl, R and Sec)

**Fig. 3:**
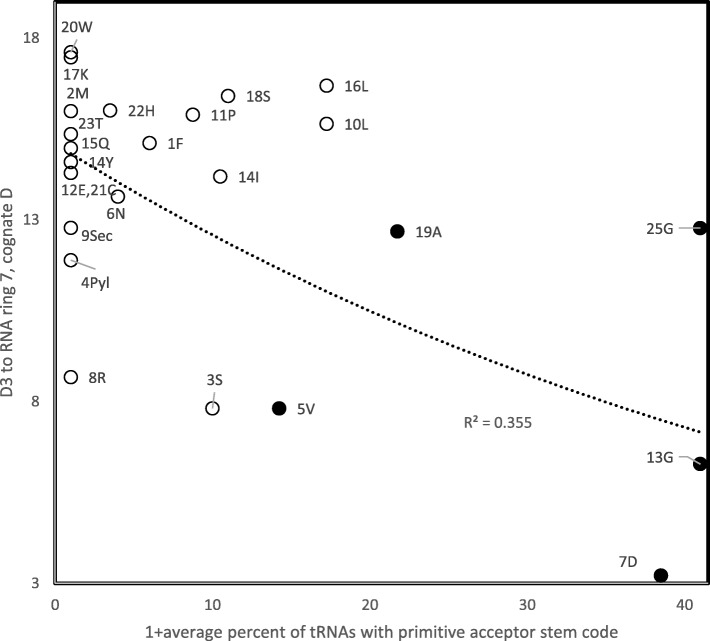
Distance D3 of RNA rings to RNA ring 7 for cognate Asp as a function of the percentage of tRNAs with a primitive code in their acceptor stem [54] (averaged across kingdoms) with the same cognate as the RNA ring. Datapoints for cognates with tRNAs considered in [54] as belonging to those with an acceptor stem primitive code (A,D,G and V) are filled, others are empty circles. The trend line indicates that D3 decreases with the presence of a primitive code in tRNA acceptor stems (*r* = − 0.596, two tailed *P* = 0.00167)

Distance D2 is a simplified approximation of known nucleotide substitution patterns. We used observed substitution rates in natural pseudogenes [61] (Table 5) to estimate D3. D3 should produce better associations than D2 because D3 should reflect observed spontaneous physicochemical substitution rates, as indicated by their proportionality with differences in nucleotide dipole moments [62, 63], because pseudogenes are presumably not functional protein coding genes. A major difference between D1 and D2, vs D3, is that D1 and D2 matrices have a diagonal with distance “0 “. This is not the case for D3, because identity rates, X- > X, differ from “1″. D3 (Table 6) associates positively with ranks of amino acid insertion in the genetic code in 58.1% of the tests. Associations were statistically significant at *P* < 0.05 (two tailed tests) for 158 cases, among which 89.9% were positive correlations. The strongest association is between D3 from RNA ring 13, and the primitive acceptor stem code (*r* = 0.63, one tailed *P* = 0.00037). RNA ring 13 is the barycenter of the sequence space formed by the 25 RNA rings, and its predicted anticodon matches glycine. Hence, it is a likely candidate for the most primitive RNA ring. The heatmap in Fig. 2 shows that D3 recovers the classification of tRNAs according to the primitive acceptor stem code. The correlation analysis in Fig. 3 confirms this, and shows the association between D3 and percentages of tRNAs with a primitive code in their acceptor stem is statistically significant (*r* = − 0.5958, two tailed *P* = 0.00167).

**Table 5:** Substitution rates between nucleotides for pseudogenes and protein coding genes as from [61], therein Table 1. XX rates subtract 1-XY rates where Y differs from X, as for AA: 1–0.030093-0.034722-0.06716 = 0.868056

X- > Y	Pseudo	CDs
AT	0.030093	0.007634
AC	0.034722	0.01145
AG	0.06713	0.020356
TA	0.02649	0.009524
TC	0.039735	0.007619
TG	0.028698	0.00381
CA	0.05026	0.014556
CT	0.133449	0.016012
CG	0.02773	0.02329
GA	0.097808	0.037037
GT	0.042159	0.008642
GC	0.033727	0.019753
AA	0.868056	0.96056
TT	0.905077	0.979048
CC	0.788562	0.946143
GG	0.826307	0.934568

**Table 6:** D3 evolutionary distances among RNA rings, using frequencies of substitutions as observed in pseudogenes [61]

D3	1	2	3	4	5	6	7	8	9	10	11	12	13	14	15	16	17	18	19	20	21	22	23	24	25
1	3.21	9.73	15.91	14.40	15.90	14.21	15.22	15.79	13.72	16.60	13.48	11.97	15.12	15.80	14.11	11.95	12.75	15.87	16.79	12.86	11.19	12.08	14.32	15.95	15.07
2	9.73	3.21	15.11	17.68	15.09	14.63	15.98	16.00	13.73	17.60	12.56	11.89	15.87	16.14	15.90	11.75	13.28	17.66	15.79	14.31	9.38	9.38	15.16	17.60	15.26
3	15.96	15.22	3.21	13.47	4.73	13.49	7.81	7.31	14.19	14.89	15.11	11.99	6.26	14.11	14.98	15.92	17.45	15.67	11.98	19.14	11.99	14.49	14.61	15.42	8.83
4	14.41	17.70	13.54	3.21	12.02	12.80	11.97	12.74	13.77	13.13	15.96	15.93	11.24	15.76	15.69	16.77	16.02	14.66	12.72	15.21	16.73	17.76	15.16	12.02	11.11
5	15.94	15.20	4.73	11.95	3.21	12.76	7.81	7.31	14.19	14.89	15.11	12.72	4.74	14.88	14.21	15.89	17.42	15.67	13.50	19.12	12.72	14.49	14.61	15.42	10.35
6	14.40	14.57	13.38	12.79	12.73	3.21	13.51	12.75	13.64	16.37	15.02	15.11	12.71	14.68	14.98	14.27	13.41	14.84	13.38	15.22	15.91	16.06	13.53	13.59	12.52
7	15.11	15.99	7.81	11.89	7.81	13.64	3.21	8.67	12.78	15.64	15.89	14.29	6.28	14.19	14.97	16.69	17.47	16.41	12.68	17.61	14.29	16.01	15.36	14.59	12.77
8	15.83	16.03	7.23	12.75	7.23	12.77	8.58	3.21	15.14	14.90	16.00	13.70	7.96	14.84	15.00	16.78	17.58	15.68	12.65	17.61	13.70	15.98	12.94	13.68	12.21
9	13.72	13.74	14.29	13.63	14.29	13.68	12.87	15.15	3.21	16.85	17.72	15.92	14.29	15.15	18.45	17.70	17.71	16.85	15.19	16.02	15.13	14.41	17.94	17.81	12.84
10	16.68	17.67	15.00	13.14	15.00	16.40	15.73	15.07	16.87	3.13	13.55	15.78	15.73	9.56	11.75	13.60	14.25	6.19	15.68	15.02	15.92	17.77	14.27	16.52	12.54
11	13.52	12.65	15.03	16.02	15.03	15.12	15.93	15.93	17.74	13.57	3.13	14.19	14.31	12.89	8.78	6.19	9.24	14.37	16.77	11.08	14.98	15.83	10.27	13.48	15.97
12	11.89	11.97	11.97	15.75	12.62	15.13	14.26	13.65	16.02	15.73	14.11	3.21	14.15	17.56	13.40	14.11	12.59	15.81	17.48	14.25	6.64	9.74	13.55	13.74	11.24
13	15.17	15.97	6.26	11.18	4.74	12.75	6.28	8.06	14.19	15.64	14.37	14.25	3.21	14.10	14.95	15.15	16.68	16.41	12.73	17.59	14.25	16.01	14.61	15.42	11.88
14	15.93	16.11	14.23	15.79	15.09	14.69	14.22	14.98	15.10	9.56	12.94	17.53	14.28	3.13	14.29	14.52	16.70	12.62	14.08	15.78	17.52	16.76	12.07	14.27	13.38
15	14.19	15.83	14.99	15.78	14.13	15.01	14.96	14.97	18.45	11.78	8.76	13.57	14.86	14.23	3.13	11.82	13.34	10.25	16.62	14.25	12.65	16.82	11.91	14.36	15.91
16	11.99	11.87	15.82	16.80	15.80	14.36	16.71	16.70	17.72	13.59	6.19	14.19	15.08	14.44	11.84	3.13	6.18	12.86	16.77	9.55	14.20	15.04	11.81	15.03	16.75
17	12.78	13.39	17.35	16.04	17.33	13.59	17.48	17.48	17.75	14.31	9.24	12.66	16.61	16.70	13.36	6.18	3.13	13.59	16.00	6.50	12.68	14.29	14.86	13.52	17.51
18	15.93	17.69	15.77	14.67	15.77	14.87	16.49	15.84	16.87	6.19	14.33	15.82	16.49	12.62	10.23	12.85	13.50	3.13	14.92	15.02	15.17	17.80	15.05	17.30	14.07
19	16.77	15.75	11.98	12.81	13.50	13.49	12.61	12.68	15.10	15.65	16.93	17.44	12.72	14.16	16.64	16.93	16.07	14.90	3.13	16.07	15.73	16.49	17.63	16.91	12.73
20	12.78	14.24	19.08	15.26	19.07	15.24	17.55	17.55	16.05	15.21	11.08	14.33	17.54	15.85	14.30	9.55	6.50	15.21	16.00	3.13	14.34	15.02	15.00	13.52	18.40
21	11.09	9.38	11.97	16.62	12.62	16.00	14.26	13.65	15.22	15.78	14.98	6.64	14.15	17.49	12.56	14.17	12.65	15.05	15.77	14.31	3.21	8.83	15.27	15.45	12.11
22	11.91	9.38	14.47	17.83	14.47	16.07	16.00	15.90	14.35	17.76	15.90	9.75	16.00	16.70	16.87	15.09	14.35	17.84	16.56	15.10	8.82	3.21	16.82	15.41	15.27
23	14.35	15.24	14.61	15.08	14.61	13.54	15.27	13.01	17.94	14.08	10.23	13.63	14.61	11.97	11.81	11.79	14.84	14.89	17.49	14.89	15.35	16.82	3.21	7.81	13.66
24	15.89	17.67	15.41	12.01	15.41	13.69	14.51	13.67	17.89	16.39	13.34	13.80	15.41	14.25	14.16	14.92	13.51	17.20	16.88	13.51	15.52	15.41	7.81	3.21	16.08
25	15.18	15.29	8.89	10.95	10.41	12.60	12.75	12.27	12.90	12.51	15.95	11.24	11.94	13.43	15.79	16.76	17.43	14.04	12.64	18.20	12.03	15.28	13.74	16.01	3.21

D3 in Table 6 includes distances for the diagonal (distances of a sequence to itself after replication), while analyses with D1 and D2 did not include this distance, which is “0”. In order to enable comparing strengths of associations of genetic code integration hypotheses with distances in Tables 4 and 6, correlations were recalculated with “0” as the distance in diagonal of Table 4. The bias for positive correlations for D2 remained very low (50.37%). Correlations were more positive for D3 than D2 in 86.9% of the comparisons. Hence, observed substitution rates reflect better RNA ring evolution than more or less arbitrary distances.

### Spontaneous physico-chemical mutations vs after natural selection

Analyses in the previous section use estimates of sequence distances derived from observed nucleotide substitutions in pseudogenes, which seem to reflect mainly neutral evolution, apparently driven by nucleotide physicochemical properties. Table 5 shows also substitution rates estimated for protein coding genes, which integrate effects of selection on substitution rates. Selection overall weeds out substitutions that tend to cause replacements between amino acids with very different properties (example glycine<− > tryptophan), disproportionally conserving nucleotide substitutions that replace amino acids by other amino acids with very similar properties (example leucine<− > isoleucine). We calculated based on these selection substitution rates D4 and a further distance matrix (not shown). Associations with genetic code integration ranks of amino acids were more positive for D3 than for D4 in 78.2% of the cases. This suggests that RNA ring evolution would have occurred without effects of selection for conserving protein coding properties of RNA rings. It potentially means that RNA ring evolution occurred under prebiotic conditions devoid of natural selection ruled by physicochemical factors, though selection for properties other than conservation of protein coding properties, such as secondary structure formation, could have driven RNA ring evolution.

A further alternative hypothesis is that similarities in codon usages of RNA rings produced a clustering compatible with the tRNA acceptor stem primitive code. We calculated D5, a matrix of similarities between RNA rings according to codon usages. Associations between genetic code integration hypotheses and D5 are much weaker than with D3, excluding that results are due to confounding effects by codon usages.

## Discussion

Comparisons between strengths of associations between genetic code inclusion order hypotheses and D1 vs D2 distances among RNA rings imply that ancient RNA rings arose spontaneously, perhaps by template-free polymerization [50, 64], and that recent RNA rings evolved from these earlier RNA rings. RNA ring properties examined in earlier analyses [23, 29, 31, 32, 41, 52, 53] coevolve with genetic code inclusion orders of their predicted cognate amino acid. This would mean that RNA rings, despite their in silico design along rational constraints, mimick the evolution of prebiotic and early life biomolecules. Indeed, observations reported here show that RNA ring clusters and distances among them match the evolution of the genetic code’s amino acid inclusions. Patterns are strengthened when considering substitution rates as observed in genes, especially pseudogenes as compared to functional protein coding genes, suggesting that RNA ring evolution was driven by physicochemical propensities for nucleotide substitutions, without effects of natural selection against substitutions causing drastic effects at amino acid replacement level. Apparently, RNA ring comparisons match, at least in part, what could be expected if RNA rings with recent cognates evolved by point mutations from those with ancient cognates. These patterns suggest that the hypothetical evolutionary diversification of RNA rings occurred without coding constraints, either because RNA rings were not translated, or because their evolution was disconnected from the function of the coded peptides.

**Fig. 4:**
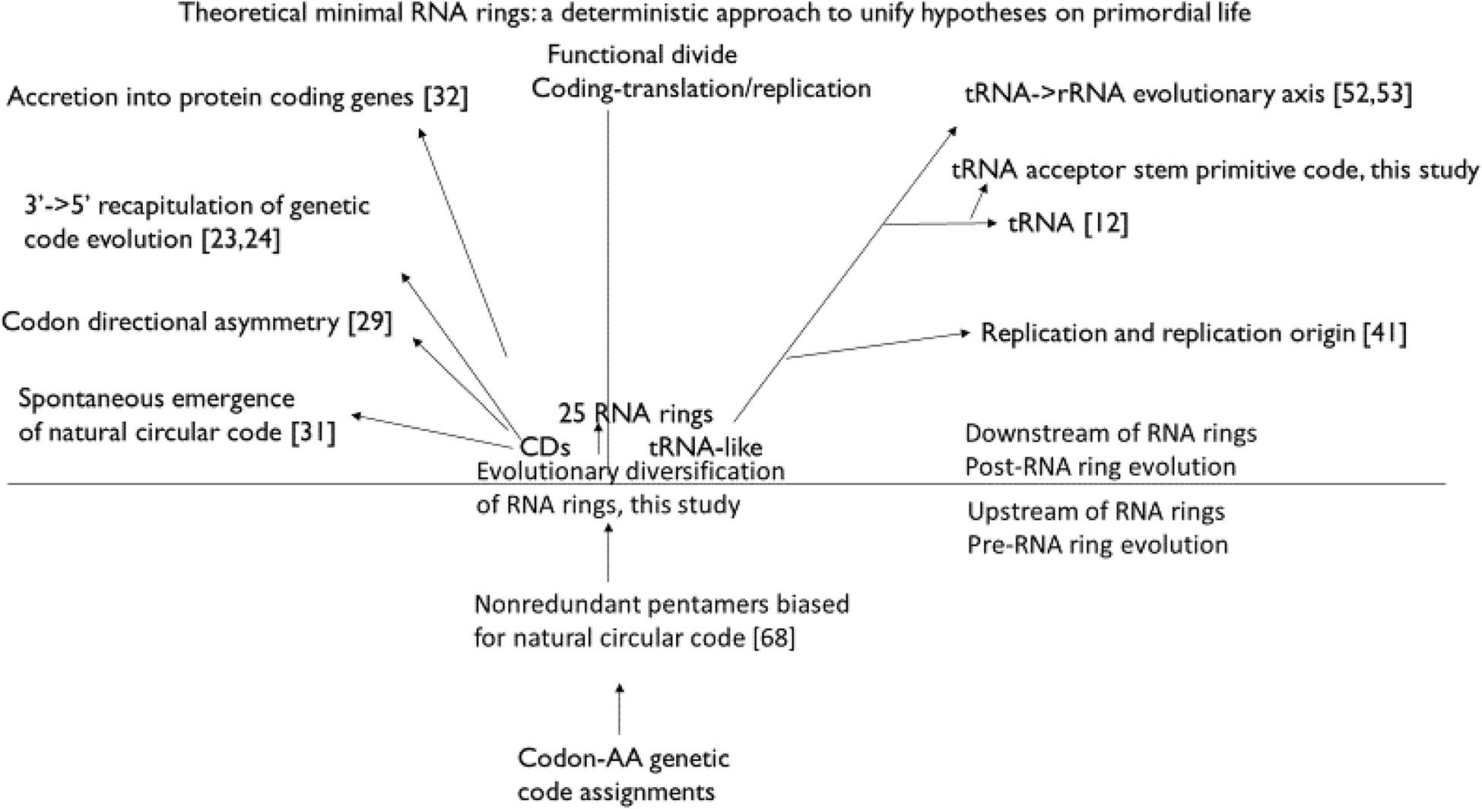
Schematic representation of prebiotic and early life evolution centered around RNA rings

Previously published analyses show how RNA ring properties mimick properties of modern genes (part above horizontal line in the scheme in Fig. 4: left of vertical line, protein coding genes; right of vertical line, structural RNAs involved in translation and replication). These observations assess the status of the RNA ring system as a system able to “compute “(or simulate/mimick) prebiotic and early life evolution of major biomolecules, by dealing with ulterior evolution, that presumably occurred downstream of RNA rings. RNAs are probably easier to use for solving RNA-related problems [65] than other problems [66], including origins of life.

This study, and most of our future endeavours, explore evolution upstream of RNA rings, corresponding to the area below the horizontal line in Fig. 4. This consists of processes that produced the RNA rings, from the presumably stereochemical determination of codon-amino acid assignments [67], and biases for short pentamers with nonredundant coding across frames [68] which could have accreted into RNA rings. The aim is to understand what makes RNA rings such useful, and perhaps efficient, simulators of prebiotic evolution, independently of the possibility that RNA rings are the actual primordial sequences.

## Conclusions

Distances among theoretical minimal RNA rings converge most with the genetic code integration hypothesis of amino acids derived from the primitive code in tRNA acceptor stems. This convergence is greatest when distances are calculated using the substitution model derived from nuclear pseudogenes. Other substitution models, such as models integrating effects of natural selection on amino acid replacements, produce weaker patterns. Hence, theoretical RNA rings evolved along physicochemical constraints affecting nucleotide substitutions, apparently devoid of effects on their coding properties on amino acid sequences, in line with a pre-translational origin of diversification of RNA rings that would at a later stage become the population of primordial coding and decoding RNAs. The RNA ring system appears as a useful synthetic simulator of prebiotic evolution [69]. This and future analyses focus on decomposing the processes and properties of RNA rings that make RNA rings such computational tools.

## Methods

The 25 theoretical minimal RNA rings are aligned considering their coding frame as presented in Table 1. This alignment does not enable insertions and/or deletions. Each RNA ring has a candidate cognate amino acid defined by its presumed anticodon [12], as it was predicted from similarities with ancestral tRNAs [1, 2]. All pairwise distances among RNA ring nucleotide sequences are calculated according to 5 models, D1-D5. The model for D1 considers that for identical nucleotides in an alignment distance “0”, and distance “1” when the presumed homologous nucleotides in Table 1 differ. D1 is the sum of the distances calculated along the complete alignment length, which is 22 nucleotide long. The model for D2 considers that when nucleotides that differ in the alignment would result from transitions, the distance should be considered as “0.5”. This is the case when the nucleotides are both purines (A and G), because these two nucleotides are relatively similar. Similarly, when both different nucleotides in the alignment are pyrimidines (C and T/U), the distance should be “0.5”. For pairs of nucleotides that would result from transversions (A < ->C, A < ->T/U, C < ->G and G < ->T/U), the distance is “1” as for D1. Table 2 presents the alignment between two specific RNA rings (13 and 25) and the calculations of D1 and D2 between this pair of RNA rings.

The model for D3 develops the principle that distances between non-identical nucleotides differ according to which nucleotide pair is considered. It is based on frequencies of observed nucleotide substitutions in pseudogenes, presumably neutrally evolving sequences. Model D4 uses also observed substitution frequencies for estimating distances between specific nucleotide pairs, but as these were observed for protein coding genes, meaning that substitution frequencies are affected by natural selection due to constraints on protein function. Empirical data on substitution frequencies for D3 and D4 are from Gojobori et al. [61]. Note that in models D3 and D4, identical nucleotides do not get distance “0”, and the distance varies according to which nucleotide is conserved, reflecting the mutability of that nucleotide.

The model for D5 compares codon usages of RNA rings and tests whether patterns of evolution could be confounded by similarities between RNA rings in codon usages, independently from the exact sequence alignment in Table 1. In this context, codon usage of each RNA ring gets value “0 “for a codon if it is not used in that RNA ring, and value “1 “if it is used in that ring. These data were used to calculate pairwise similarities among all combinations of two RNA rings, using Pearson’s correlation coefficient r. These correlation coefficients are similarities, with the maximal similarity at *r* = 1. In order to obtain a distance, we used 1-r as distance D5. The corresponding D5 matrix is used in calculations, as are distance matrices D1-D4.

For each distance matrix D1 to D5, further analyses consider separately each row of the matrix, which corresponds to distances from a given focal RNA ring to each of the remaining “target” RNA rings. Correlations between each of these 25 sets of distances and the genetic code integration order of the cognate amino acid of the target RNA ring are calculated. This is done for each of the genetic code integration hypotheses listed by Trifonov [51]. Overall, results confirm that distances increase with the genetic code integration rank, and this trend is strongest for D3, as compared to all other distances D1, D2, D4 and D5. Specifically, the genetic code evolutionary hypothesis that produces the strongest associations between the various distances (D1–5 confounded) is the evolutionary hypothesis derived from the primitive code in tRNA acceptor stems [54].

## Data Availability

The datasets supporting the conclusions of this article are included within the article.

## Abbreviations

A: One letter abbreviation for amino acid alanine
C: One letter abbreviation for amino acid cysteine
CD: Coding sequence
CDA: Codon directional asymmetry
D: One letter abbreviation for amino acid aspartic acid
D1: Hamming distance between RNA rings
D2: Hamming evolutionary distance between RNA rings distinguishing transitions and transversions
D3: Evolutionary distances among RNA rings, using frequencies of substitutions as observed in pseudogenes
D4: Evolutionary distances among RNA rings, using frequencies of substitutions as observed in protein coding genes
D5: Distance between RNA rings based on differences in codon usages
E: One letter abbreviation for amino acid glutamic acid
F: One letter abbreviation for amino acid phenylalanine
G: One letter abbreviation for amino acid glycine
GNN: Codons with G at position 1
H: One letter abbreviation for amino acid histidine
I: One letter abbreviation for amino acid isoleucine
K: One letter abbreviation for amino acid lysine
L: One letter abbreviation for amino acid leucine
M: One letter abbreviation for amino acid methionine
N: One letter abbreviation for amino acid asparagine
P: One letter abbreviation for amino acid proline
Pyl: Abbreviation for amino acid pyrrolysine
Q: One letter abbreviation for amino acid glutamine
R: One letter abbreviation for amino acid arginine
RNY: Codons a purine (R), any nucleotide (N) and a pyrimidine (Y) at positions 1, 2, and 3, respectively.
S: One letter abbreviation for amino acid serine
Sec: Abbreviation for amino acid selenocysteine
T: One letter abbreviation for amino acid threonine
V: One letter abbreviation for amino acid valine
W: One letter abbreviation for amino acid tryptophan
Y: One letter abbreviation for amino acid tyrosine
